# Assembly of flexible CoMoO_4_@NiMoO_4_·xH_2_O and Fe_2_O_3_ electrodes for solid-state asymmetric supercapacitors

**DOI:** 10.1038/srep41088

**Published:** 2017-01-20

**Authors:** Jing Wang, Leipeng Zhang, Xusong Liu, Xiang Zhang, Yanlong Tian, Xiaoxu Liu, Jiupeng Zhao, Yao Li

**Affiliations:** 1 MIIT Key Laboratory of Critical Materials Technology for New Energy Conversion and Storage, School of Chemistry and Chemical Engineering, Harbin Institute of Technology, Harbin 150001, PR China; 2 Center for Composite Materials, Harbin Institute of Technology, Harbin 150001, PR China; 3 Heilongjiang University of Science and Technology, Harbin 150022, PR China

## Abstract

In this work, CoMoO_4_@NiMoO_4_·xH_2_O core-shell heterostructure electrode is directly grown on carbon fabric (CF) via a feasible hydrothermal procedure with CoMoO_4_ nanowires (NWs) as the core and NiMoO_4_ nanosheets (NSs) as the shell. This core-shell heterostructure could provide fast ion and electron transfer, a large number of active sites, and good strain accommodation. As a result, the CoMoO_4_@NiMoO_4_·xH_2_O electrode yields high-capacitance performance with a high specific capacitance of 1582 F g^−1^, good cycling stability with the capacitance retention of 97.1% after 3000 cycles and good rate capability. The electrode also shows excellent mechanical flexibility. Also, a flexible Fe_2_O_3_ nanorods/CF electrode with enhanced electrochemical performance was prepared. A solid-state asymmetric supercapacitor device is successfully fabricated by using flexible CoMoO_4_@NiMoO_4_·xH_2_O as the positive electrode and Fe_2_O_3_ as the negative electrode. The asymmetric supercapacitor with a maximum voltage of 1.6 V demonstrates high specific energy (41.8 Wh kg^−1^ at 700 W kg^−1^), high power density (12000 W kg^−1^ at 26.7 Wh kg^−1^), and excellent cycle ability with the capacitance retention of 89.3% after 5000 cycles (at the current density of 3A g^−1^).

In recent years, smart electronic devices have attracted tremendous research interest due to their unique features and potential applications in the next generation wearable electronic devices^1^,^2^,^3^. However, meeting the increasing demand of future emerging markets are lightweight, solid state, and flexible energy devices^4^,^5^,^6^,^7^. Among them, supercapacitors (SCs) are considered as promising candidates that can offer long cycle life, high power density, fast charge/discharge rates and safety^8^,^9^,^10^,^11^,^12^,^13^,^14^,^15^. Unfortunately, the energy densities of supercapacitors are still unsatisfactory, which seriously limit their practical applications. According to the energy density equation: *E* = 1/2 *CV*^*2*^ 16,17, the energy density (*E*) can be enhanced by improving the specific capacitance (*C*) and/or the operating potential window (*V*). Therefore, an effective alternative approach to increase the energy density is to fabricate the asymmetric supercapacitors (ASC). It can make full use of the different potential windows of the two electrodes to provide a maximum operation voltage in the cell system, accordingly resulting in a greatly enhanced specific capacitance and significantly improved energy density.

Recently, a variety of materials have been explored for possible applications as the cathode in asymmetric supercapacitors, such as transition metal oxides, metal hydroxides and conducting polymer^18^,^19^,^20^,^21^,^22^,^23^. In particular, metal molybdates are particularly attractive as an important family of inorganic materials^24^.

Previous studies show that CoMoO_4_ has excellent rate capability and cyclic properties. However, its specific capacitance is lower than that of most reported oxides^25^. Interestingly, NiMoO_4_ possesses a high specific capacitance, ascribed to the high electrochemical activity of the nickel ion, but its rate capability is inferior. This encouraged some researchers to fabricate a CoMoO_4_ and NiMoO_4_ based composite with a unique nanostructure, which will combine the advantages of both CoMoO_4_ and NiMoO_4_. For example, Liu *et al*. synthesized CoMoO_4_-NiMoO_4_·xH_2_O bundles on Ni foam by a chemical co-precipitation method. The mixed bundles showed a capacitance of 1039F g^−1^ at a current density of 1A g^−1^ and excellent rate capability, superior to single-phase NiMoO_4_·xH_2_O^26^. Yin *et al*. reported active carbon and hierarchical nanosheet-based CoMoO_4_-NiMoO_4_ nanotubes as negative and positive electrodes of the supercapacitor, respectively. This electrode delivered high specific energy of 33 W h kg^−1^ and high power density of 6000 W kg^−1^ 27. Zhang and his coworkers showed NiMoO_4_@CoMoO_4_ hierarchical nanospheres and explored the symmetric capacitor with the potential window of 1.5 V^28^.

The above reports indicate that the composite materials with special structures show better performance than those of the individual component. Therefore, this motivated us to design and fabricate an elegant CoMoO_4_ and NiMoO_4_ core-shell heterostructured electrode with a novel nanostructure, which might combine the merits of both high specific capacitance originating from NiMoO_4_ and excellent rate capability provided by CoMoO_4_, together with the well-designed architecture to improve the performance.

At present, carbonaceous materials are still the most mainly used anode materials, such as activated carbon, grapheme, and nitrogen-doped carbons^29^,^30^,^31^,^32^. However, carbonaceous materials show quite small specific capacitances (100–250F g^−1^) compared with pseudocapacitive anode^33^,^34^,^35^,^36^. Among the reported pseudocapacitive anode materials, Fe_2_O_3_ exhibits many advantages such as low cost, environmental friendliness and safety^37^,^38^,^39^,^40^. Moreover, Fe_2_O_3_-based electrode shows higher specific capacitance and wider voltage window compared with the carbonaceous materials. However, the intrinsic poor conductivity and aggregated morphology of Fe_2_O_3_-based electrodes restrict their electrochemical performance. An effective method is to synthesize the binder-free electrodes, which directly grown on the conductive substrates. Hence, the poor conductivity of the Fe_2_O_3_ electrode is no longer a problem. And the nanostructures facilitate the electrolyte to penetrate the inner part of electrodes and benefits for improving the utilization of their pseudocapacitance^41^.

In this work, we report the flexible CoMoO_4_@NiMoO_4_·xH_2_O core-shell heterostructures cathode and Fe_2_O_3_ nanorods (NWs) anode directly grown on the carbon fabric for high-performance asymmetric solid-state supercapacitors. As a cathode material, the flexible 3D networked CoMoO_4_@NiMoO_4_·xH_2_O core-shell heterostructures electrode exhibits excellent electrochemical performance in contrast to that of individual flexible CoMoO_4_ and NiMoO_4_·xH_2_O. It shows a high capacitance of 1582F g^−1^, high rate performance, as well as an outstanding long-term cycling ability with the capacitance retention of 97.1% after 3000 cycles at 1A g^−1^. Even at the current density of 5A g^−1^, the capacitance still can be retained up to 93.2% after 10000 cycles. As an anode material, the flexible Fe_2_O_3_ nanorods electrode exhibits the specific capacitance of 516.7F g^−1^ at the current density of 1A g^−1^, which is much higher than carbonaceous materials. Even at a high current density of 15A g^−1^, it still retains a specific capacitance of 312.5F g^−1^. In addition, since the negative potential window for the Fe_2_O_3_ nanorods is 0~−1.2 V, the Fe_2_O_3_ nanorods anode and the CoMoO_4_@NiMoO_4_·xH_2_O cathode can fully utilize their large theoretical pseudocapacitance in the corresponding complementary potential windows. Therefore, the perfect matching between CoMoO_4_@NiMoO_4_·xH_2_O and Fe_2_O_3_ nanorods is quite obvious. The solid-state asymmetric CoMoO_4_@NiMoO_4_·xH_2_O//Fe_2_O_3_ supercapacitor device with a maximum voltage of 1.6 V shows high specific energy (41.8 Wh kg^−1^ at 700 W kg^−1^), power density (12000 W kg^−1^ at 26.7 Wh kg^−1^) and excellent cycling stability.

## Results and Discussion

**Figure 1:**
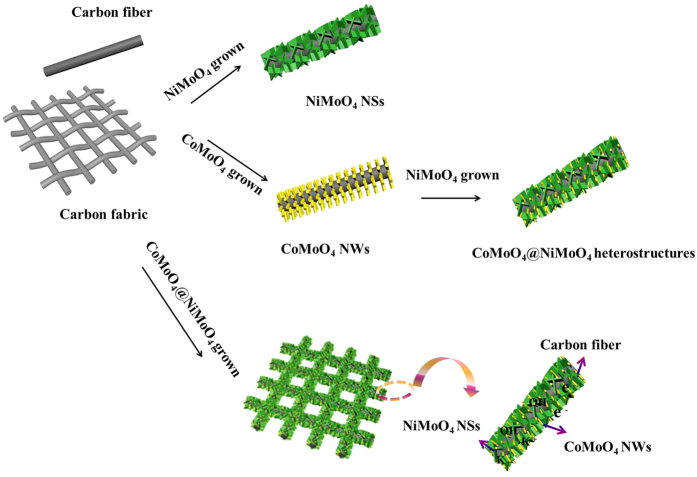
Schematic illustration for the fabrication of the flexible CoMoO_4_@NiMoO_4_·xH_2_O core-shell heterostructures.

**Figure 2:**
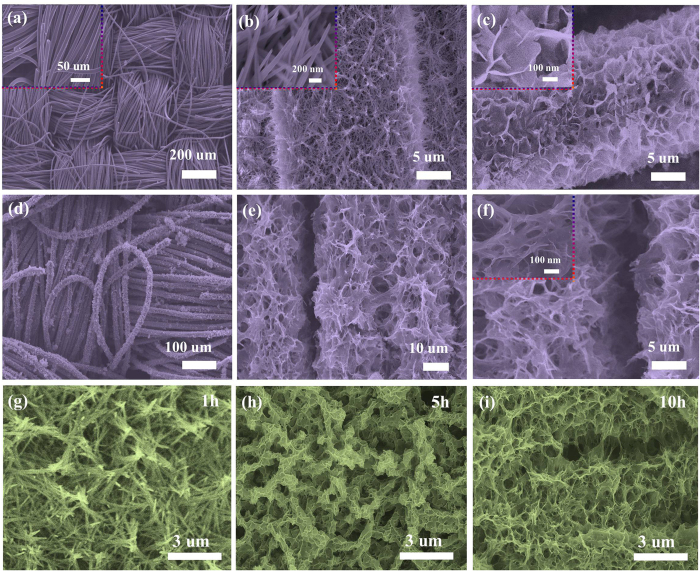
SEM images of (**a**) Carbon fabric; (**b**) CoMoO_4_ NWs on carbon fabric. (**c**) NiMoO_4_**·**xH_2_O NSs on carbon fabric. (**d**–**f**) Flexible CoMoO_4_@NiMoO_4_**·**xH_2_O core-shell heterostructures taken at different magnifications. The insets are the high magnification SEM images. (**g**–**i**) SEM images of the CoMoO_4_@NiMoO_4_·xH_2_O at different reaction times from 1 h to 10 h.

The schematic illustration for the preparation of the CoMoO_4_@NiMoO_4_·xH_2_O core-shell heterostructure grown on carbon fabric is presented in Fig. 1. CoMoO_4_@NiMoO_4_·xH_2_O composites were prepared by a simple template-free hydrothermal process coupled with a calcination treatment. The formation schematic illustration of CoMoO_4_@NiMoO_4_·xH_2_O composites grown on carbon fiber (CF) was presented in Fig. 1. The preparation process mainly involves two steps. In the first step, a light purple CoMoO_4_ precursor is generated on the carbon cloth surface by hydrothermal reaction. After heat treatment, the dark purple CoMoO_4_ NWs were supported on the carbon cloth. In the second step, CoMoO_4_ NWs were immersed into the light green precursor solution of NiMoO_4_ for further hydrothermal process and heat treatment. Finally, flexible CoMoO_4_@NiMoO_4_·xH_2_O composites were formed on the CF. Optical images of the as prepared electrodes are shown in Fig. S1. The morphologies and microstructures of the as prepared products were investigated and the results are shown in Fig. 2. The SEM image in Fig. 2a shows the carbon fabric composed of crossed carbon fibers with the average diameter of about 15 μm. The morphology of the CoMoO_4_ NWs is shown in Fig. 2b, which indicates the products with high density are uniformly distributed on the fibers of the CF. The CoMoO_4_ NWs have an average diameter of 100 nm and length of around 1.5 μm. Figure 2c reveals the SEM image of NiMoO_4_·xH_2_O NSs which possesses a nanostructure composed of nanosheets with an average thickness of 10 nm. These nanosheets are interconnected with each other and contain a highly porous network structure. Figure 2d indicates the final product, the networked CoMoO_4_@NiMoO_4_·xH_2_O nanostructures are successfully produced on the carbon fibers on a large scale. Figure 2e,f clearly demonstrates that NiMoO_4_·xH_2_O NSs are homogeneously covered on the whole surfaces of CoMoO_4_ NWs, forming an interconnected and a highly porous 3D morphology, which may offer not only 3D networks for fast electron transportation, but also spaces critical for ion diffusion. The experiments with different reaction times changed from 1 h to 10 h were further researched to explore the composite structure as shown in Fig. 2g–i. When the reaction time is 1 h, the morphology seems like nanowires without NiMoO_4_ NSs deposition on CoMoO_4_ NWs. When the reaction time up to 5 h, it can be found that the whole CoMoO_4_ layer was covered by NiMoO_4_ nanosheets and the whole CoMoO_4_ nanowires arrays’ morphology is remained. Further changing the reaction time to 10 h, the whole CoMoO_4_ layer was covered by much more NiMoO_4_ nanosheets has been deposited with an obvious change in morphology. The morphology is core-shell structure with much more NiMoO_4_ nanosheets deposited on CoMoO_4_ nanowires arrays. TEM images with different magnifications have been conducted (Fig. S2). It further confirmed that the CoMoO_4_ layer was covered by much more NiMoO_4_ nanosheets has been deposited. Brunauer-Emmett-Teller (BET) analysis results show that the specific surface area of CoMoO_4_@NiMoO_4_·xH_2_O is 100.79 m^2^ g^−1^, which is much higher in contrast to that of CoMoO_4_ NWs (37.93 m^2^ g^−1^) and NiMoO_4_·xH_2_O NSs (79.37 m^2^ g^−1^) (Fig. S3). This core-shell configuration can provide a higher surface area, which is mainly attributed to the interconnected NiMoO_4_·xH_2_O NSs and the aligned CoMoO_4_ NWs scaffold that creating a 3D structure and highly porous surface morphology. Such configuration is of great importance to promote electrolytes accessibility and increase the utilization of the active materials. The whole zone of Fig. 2f is selected to research the SEM mapping (Fig. S4). It can be clearly seen that only elements of O, Co, Ni and Mo could be found in CoMoO_4_@NiMoO_4_·xH_2_O.

The phase structures of the as-prepared products were analyzed by X-ray diffraction. As shown in Fig. S5, NiMoO_4_·xH_2_O and CoMoO_4_ are in good agreement with the standard patterns for NiMoO_4_·xH_2_O (PDF, card no. 13-0128) and monoclinic CoMoO_4_ (PDF, card no. 21-0868), respectively. In addition, several weak diffraction peaks attributed to the impurity phase of NiMoO_4_ (PDF, card no. 12-0348) and CoMoO_6_ ·0.9H_2_O (PDF, card no. 14-1186) are found. The results are consistent with the previous research^42^. The XRD pattern of CoMoO_4_@NiMoO_4_·xH_2_O contains the diffraction peaks of both NiMoO_4_·xH_2_O and CoMoO_4_, indicating the presence of both phases.

**Figure 3:**
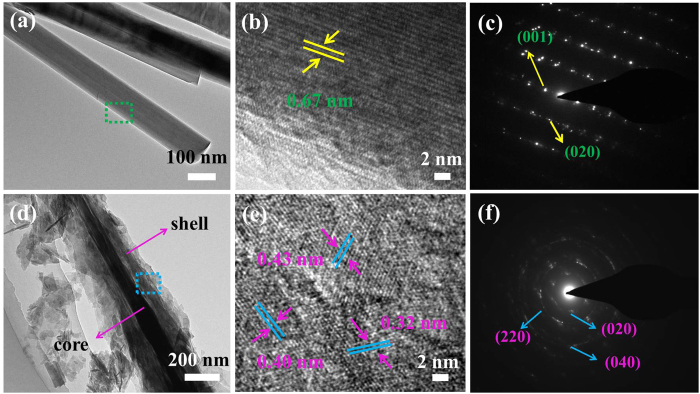
(**a**) TEM image of an individual CoMoO_4_ NW; (b) HRTEM image of the CoMoO_4_ NW; (**c**) SAED pattern of CoMoO_4_ NW; (**d**) TEM image of the CoMoO_4_@NiMoO_4_**·**xH_2_O core-shell heterostructures; (**e**) HRTEM image of the CoMoO_4_@NiMoO_4_**·**xH_2_O core-shell heterostructures; (**f**) SAED pattern of CoMoO_4_@NiMoO_4_**·**xH_2_O core-shell heterostructures.

The microstructure of the as-prepared products was further characterized by TEM and SAED. Figure 3a depicts the low-magnification TEM image of CoMoO_4_ NWs with the diameter of about 100 nm. The measured lattice spacing of 0.67 nm in HRTEM image (Fig. 3b) is corresponding to the (001) planes of monoclinic CoMoO_4_. Figure 3c shows the corresponding selected area electron diffraction (SAED) pattern. The SAED pattern of the CoMoO_4_ shows a set of well-defined spots, indicative of its single-crystallinity property. The diffraction rings can be readily indexed to the (001), and (020) planes of the CoMoO_4_ phase, which is consistent with the above XRD result. TEM images in Fig. 3d confirm the core-shell structure with the CoMoO_4_ NWs as the core parts and NiMoO_4_·xH_2_O NSs as the shell layers. HRTEM image (Fig. 3e) reveals the interplanar spacing of 0.43 nm, 0.40 nm, and 0.32 nm, corresponding to those of 4.30 Å, 4.06 Å, and 3.26 Å given in the PDF 13-0128 in the standard files of NiMoO_4_·xH_2_O^43^,^44^. The SAED pattern (Fig. 3f) indicates the polycrystalline nature of CoMoO_4_@NiMoO_4_·xH_2_O, and the diffraction rings can be readily indexed to the (020), (220) and (040) planes of the NiMoO_4_ phase, which is consistent with the above XRD result. TEM images and elemental mapping of the CoMoO_4_ and CoMoO_4_@NiMoO_4_·xH_2_O (Fig. S6) further indicate that the elements of Co, Mo, O and Ni are distributed uniformly on the core and the shell.

**Figure 4:**
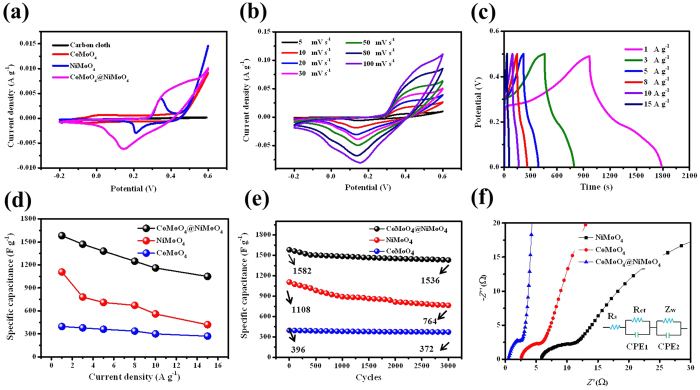
(**a**) CV curves of the CF, CoMoO_4_ NWs, NiMoO_4_**·**xH_2_O NSs, and CoMoO_4_@NiMoO_4_**·**xH_2_O core-shell heterostructures at a scan rate of 5 mV s^−1^; (**b**) CV curves of CoMoO_4_@NiMoO_4_**·**xH_2_O at the scan rate between 5 and 100 mV s^−1^; (**c**) Charge and discharge curves of CoMoO_4_@NiMoO_4_**·**xH_2_O at different current densities ranged from 1 to 15A g^−1^; (**d**) Plots of the current density against specific capacitances of the CoMoO_4_ NWs, NiMoO_4_**·**xH_2_O NSs and CoMoO_4_@NiMoO_4_**·**xH_2_O core-shell heterostructures electrodes obtained from the galvanostatic charge-discharge curves; (**e**) Cycling performance of CoMoO_4_ NWs, NiMoO_4_**·**xH_2_O NSs and CoMoO_4_@NiMoO_4_**·**xH_2_O core-shell heterostructures at a discharge current density of 1A g^−1^; (**f**) Nyquist plots of CoMoO_4_ NWs, NiMoO_4_**·**xH_2_O NSs and CoMoO_4_@NiMoO_4_**·**xH_2_O core-shell heterostructures.

The electrochemical storage application of as-prepared products was evaluated by testing them as electrodes for supercapacitors in a three-electrode configuration. We firstly compared the cyclic voltammetric (CV) curves of CF, CoMoO_4_ NWs, NiMoO_4_·xH_2_O NSs, and CoMoO_4_@NiMoO_4_·xH_2_O electrodes at the scan rate of 5 mV s^−1^ (Fig. 4a). The results indicate that the contribution of the CF substrate is tiny compared with the other three electrodes. The current density and enclosed CV curve area of the CoMoO_4_@NiMoO_4_·xH_2_O are much larger than CoMoO_4_ NWs and NiMoO_4_·xH_2_O NSs, due to the networked porous hybrid structural effect from the CoMoO_4_ NWs and ultrathin NiMoO_4_·xH_2_O NSs. Figure 4b further describes the CV characteristics of the CoMoO_4_@NiMoO_4_·xH_2_O electrode at different scan rates. Each CV curves consist of a pair of redox peaks, indicating pseudocapacitive behavior of CoMoO_4_@NiMoO_4_·xH_2_O. The Faradic reactions correspond to the redox peaks for CoMoO_4_ NWs^45^,^46^,^47^ and NiMoO_4_·xH_2_O NSs^48^,^49^ are as follows:


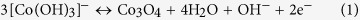



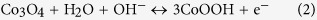



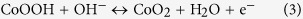



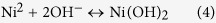



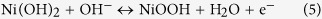


The electrochemical capacitance of CoMoO_4_@NiMoO_4_·xH_2_O is attributed to the quasi reversible electron transfer process that mainly involves the Co^2+^/Co^3+^ and the Ni^2+^/Ni^3+^ redox couple, and probably mediated by the OH^−^ ions in the alkaline electrolyte. The main function of Mo element is to improve the conductivity of metal molybdates and achieve the enhanced electrochemical capacitance^26^,^46^,^47^. The peak current increases linearly with the increase of the scan rate, which suggests that the kinetics of the interfacial Faradic redox reactions and the rates of electronic and ionic transport are rapid enough in the present scan rates. The shape of the CV curves is not significantly influenced by the increase of the scan rates, which indicates the improved mass transportation and electron conduction in the host materials. Figure 4c shows the galvanostatic charge-discharge (GCD) curves of the CoMoO_4_@NiMoO_4_·xH_2_O electrode within a potential range of 0 to 0.5 V at various current densities. The corresponding comparative CV and GCD curves of CoMoO_4_ NWs, and NiMoO_4_·xH_2_O NSs are shown in Fig. S7. The specific capacitances of the three electrodes derived from the discharging curves at different current densities are compared and shown in Fig. 4d. The specific capacitances of CoMoO_4_@NiMoO_4_·xH_2_O calculated according to the Equation (6) are 1582, 1470, 1380, 1248, 1160, and 1050F g^−1^ at the current densities of 1, 3, 5, 8, 10, and 15A g^−1^, much higher than those of the pristine CoMoO_4_ NWs and NiMoO_4_·xH_2_O NSs. This core-shell CoMoO_4_@NiMoO_4_·xH_2_O heterostructure shows a rate capability of 64% with a high specific capacitance of 1582F g^−1^ at a current density of 1A g^−1^ and 1050F g^−1^ at a current density of 15A g^−1^. The CoMoO_4_ NWs exhibits a good rate capability of 68.2% but a low specific capacitance of 396F g^−1^ at a current density of 1A g^−1^ and 270F g^−1^ at a current density of 15A g^−1^. The NiMoO_4_·xH_2_O NSs shows a high specific capacitance of 1108F g^−1^ at the current density of 1A g^−1^, but only 37.9% of this value remained at a high current density of 15A g^−1^, indicating relatively weaker rate capability compared with CoMoO_4_ NWs. Nevertheless, the CoMoO_4_@NiMoO_4_·xH_2_O combines the advantages of the good rate capability of CoMoO_4_ and the high specific capacitance of NiMoO_4_·xH_2_O. The cyclic stability of supercapacitors is another critical issue in practical use. Cyclic tests for the three electrodes were carried out for over 3000 cycles at 1A g^−1^. Figure 4e presents that the CoMoO_4_@NiMoO_4_·xH_2_O electrode exhibits an excellent long-term stability with only 2.9% capacitance loss after 3000 cycles, which is much better than 6.1% capacitance loss for the CoMoO_4_ NWs and 30% capacitance loss for the NiMoO_4_·xH_2_O NSs electrode after the same cycles. The charge/discharge curves of the CoMoO_4_@NiMoO_4_·xH_2_O electrode obtained at the last cycle are remained much the same as the ones obtained in the first cycle (Fig. S8). Furthermore, tests were also carried out for up to 10000 cycles at a current density of 5A g^−1^. As shown in Fig. S9, the CoMoO_4_@NiMoO_4_·xH_2_O electrode exhibits excellent long-term stability with 93.2% capacitance retention. In addition, the charge-discharge curves shape the insets in Fig. S9 are still keeping quite stable after 10000 cycles, indicating the CoMoO_4_@NiMoO_4_·xH_2_O electrode has good cycle performance. Figure S10 shows SEM images of the CoMoO_4_@NiMoO_4_·xH_2_O electrode before and after 10000 cycles. It shows that a few of the CoMoO_4_@NiMoO_4_·xH_2_O aggregate compared with that of the as-prepared CoMoO_4_@NiMoO_4_·xH_2_O after 10000 cycles.

To further insight into the influence of electrochemical impedance to the electrode for supercapacitors, electrochemical impedance spectroscopy (EIS) of the CoMoO_4_ NWs, the NiMoO_4_·xH_2_O NSs and the CoMoO_4_@NiMoO_4_·xH_2_O electrodes were measured in the frequency range from 0.01 Hz to 100 kHz at an open circuit potential with a superimposed 5 mV sinusoidal voltage (Fig. 4f). The three electrodes indicate similar two forms with a semicircle at the high frequency region and a straight line at the low frequency. At the high frequency, the intersection of the curve at the real part shows the resistance of the electrochemical system (R_s_) and the semicircle diameter shows the charge-transfer resistance (R_ct_). R_s_ includes the ionic resistance of electrolyte, inherent resistance of the electroactive material, and contact resistance at the interface between electrode and electrolyte^50^. The semicircle of the Nyquist plot corresponds to the Faradic reactions and its diameter represents the interfacial R_ct_ in the high frequency. The inset in Fig. 4f shows an equivalent circuit used to match with the EIS curves to measure R_s_ and R_ct_. Z_w_ and CPE are the Warburg impendence reflected by the straight line in the low frequency^51^,^52^. As expected, CoMoO_4_@NiMoO_4_·xH_2_O shows the lower internal resistances (R_s_) 0.62 Ω compared with CoMoO_4_ (2.76 Ω) and NiMoO_4_·xH_2_O (6.15 Ω), indicative of improved electrical conductivity. The CoMoO_4_@NiMoO_4_·xH_2_O electrode also demonstrates lower charge-transfer resistance 1.86 Ω than CoMoO_4_ (5.24 Ω) and NiMoO_4_·xH_2_O (8.85 Ω) as shown in Fig. 4f. Moreover, the CoMoO_4_@NiMoO_4_·xH_2_O electrode also demonstrates the smallest diffusive resistance. The above results show that the combination of fast ion diffusion as well as low electro-transfer resistance is also responsible for the enhanced electrochemical performance of the CoMoO_4_ and NiMoO_4_·xH_2_O core-shell electrode. This is mainly caused by the networked porous core-shell structure with larger specific surface area, resulting in enhanced utilization of the electrode materials and facilitated supply of OH^−^ to the electrode^53^. It is believed that the hybrid structure with low diffusion and electron-transfer resistances are beneficial to the excellent supercapacitor performance.

**Figure 5:**
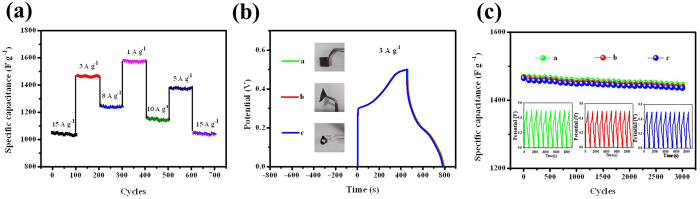
(**a**) Rate performance and cycling stability of the CoMoO_4_@NiMoO_4_**·**xH_2_O electrode under different current densities; (**b**) Charge and discharge curves of the CoMoO_4_@NiMoO_4_**·**xH_2_O electrode under bending and twisting of three different forms. The insets are the images of the CoMoO_4_@NiMoO_4_**·**xH_2_O electrode under bending and twisting of three different forms; (**c**) Cycling performance of the CoMoO_4_@NiMoO_4_**·**xH_2_O electrode at a discharge current density of 3A g^−1^ under different bending conditions for 3000 cycles. The insets are charge-discharge curves at a current density of 3A g^−1^ after the first tenth of cycles.

Figure 5a further reveals the current density dependence of the cycling performance of the CoMoO_4_@NiMoO_4_·xH_2_O electrode. A stable specific capacitance of about 1050F g^−1^ can be found in the first 100 cycles at the current density of 15A g^−1^. Changing the current density successively, the hybrid electrode still exhibits stable capacitance in different forms. When changing the current density back to 15A g^−1^, the electrode can fully recover the specific capacitance of 1050F g^−1^. These results further indicate the CoMoO_4_@NiMoO_4_·xH_2_O electrode has excellent stabilities and rate performance.

To explore the flexibility of the electrode, we compared the GCD curves and cyclic performance of the electrode under flat and bending for electrochemical test at a current density of 3A g^−1^. As indicated in Fig. 5b and c, the GCD profiles confirm the negligible attenuation of charge-discharge interval of the bent electrode compared to its flat state. Figure 5c shows the corresponding specific capacitance variation tendency under a bent state compared to its natural state after 3000 cycles. The specific capacitance retention for the flat one is 99.3% and the other two bent forms are still keeping 98.9% and 98.5% capacitance, respectively. The corresponding GCD curves of the first ten cycles for the three forms show no obvious changes (the insets). The results further confirm the electrode is mechanically robust.

The high specific capacitance of the CoMoO_4_@NiMoO_4_ core-shell heterostructures electrode are impressive values when compared to those of many previously reported CoMoO_4_ or NiMoO_4_ oxides based electrodes, as shown in Table S1. The above results reveal high specific capacity, excellent cycling stability, outstanding rate capability, and mechanically flexibility of the CoMoO_4_@NiMoO_4_·xH_2_O core-shell electrode. It mainly attribute to the 3D networked heterostructure and a direct growth on the flexible conductive carbon fabric substrate. As schematically demonstrated in Fig. 1, first, carbon fabric as conductive substrate has good electrical conductivity, high porosity and excellent mechanical flexibility. This leads to the fact that electrons can transport more efficiently during charge-discharge processes and therefore large improvement in specific capacitance. Second, the unique core-shell hierarchical structure has an increased portion of exposed surface, which provides more active sites for ions and electrons access to the surface of the electrode. The porosity of the surface further shortens the diffusion paths for ions so that accelerate the redox reaction to take place and enhance the rate capability. Thirdly, this 3D networked core-shell nanostructures on carbon fabric is a stale architecture with excellent mechanical robust and flexibility, which can improve the cycling stability evidently during long-term cycling. Finally, the CoMoO_4_ and NiMoO_4_·xH_2_O are good pseudocapacitor materials due to their multiple oxidation states for reversible Faradaic reactions. The heterostructure allows synergistic contributions from the CoMoO_4_ with excellent cycling ability and good rate capability, and NiMoO_4_·xH_2_O with high specific capacitance.

In order to research the practical application of the as-prepared electrodes, flexible solid-state asymmetric supercapacitor device was assembled by using CoMoO_4_@NiMoO_4_·xH_2_O core-shell heterostructures as the cathode and the Fe_2_O_3_ NRs as anode, respectively. Before assembling the asymmetric supercapacitor device, we firstly researched the microstructure and electrochemical properties of Fe_2_O_3_ NRs. Figure S11a shows SEM image of the as synthesized Fe_2_O_3_ NRs with an average diameter of 100 nm and length of approximate 200 nm directly attached to the CF substrate. The phase structures of the as-prepared Fe_2_O_3_ NRs were analyzed by X-ray diffraction. Figure S11b indicates the as-prepared Fe_2_O_3_ is in good agreement with the standard pattern for rhombohedral Fe_2_O_3_ (PDF, card no. 33-0664). To investigate the electrochemical performance of the Fe_2_O_3_ nanorods, we tested the CV curves at different scan rates in a three-electrode measurement (Fig. S12a). Figure S12b exhibits the GCD curves at different current densities with the potential window 0~−1.2 V and the specific capacitances are calculated from the GCD curves. At the current density of 1A g^−1^, the Fe_2_O_3_ nanorods exhibit the specific capacitance of 516.7F g^−1^. Even at a high current density of 15A g^−1^, it can still retain a specific capacitance of 312.5F g^−1^. The unique 1D nanostructure is quite beneficial for the rapid electrolyte flow to more accessible electrochemical active sites, enhancing the capacitive performance as a result. The electrochemical performance of the Fe_2_O_3_ NRs in the wide negative potential window and high specific capacitance are favorable for using as an anode material.

Based upon the above experimental results and discussions, the perfect matching between the flexible CoMoO_4_@NiMoO_4_·xH_2_O and Fe_2_O_3_ NRs electrodes is quite obvious. The Fe_2_O_3_ NRs anode and the CoMoO_4_@NiMoO_4_·xH_2_O cathode can fully utilize their large theoretical pseudocapacitance in the corresponding complementary potential windows. As shown in Fig. S13, they exhibit large pseudocapacitance in the exactly complementary potential windows. The charge balances for the positive and negative electrodes have been calculated in the supporting information.

**Figure 6:**
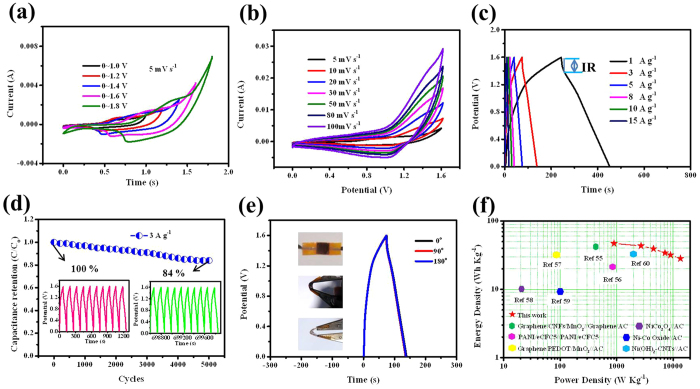
(**a**) CV curves of the CoMoO_4_@NiMoO_4_**·**xH_2_O//Fe_2_O_3_ ACS device coll ected at different potential voltages at a scan rate of 5 mV s^−1^; (**b**) CV curves of the CoMoO_4_@NiMoO_4_**·**xH_2_O//Fe_2_O_3_ ASC device collected at various scan rates; (**c**) Charge-discharge curves of the CoMoO_4_@NiMoO_4_**·**xH_2_O//Fe_2_O_3_ ASC device collected at various current densities; (**d**) Cycling performance of the CoMoO_4_@NiMoO_4_**·**xH_2_O//Fe_2_O_3_ ACS device at a discharge current density of 3A g^−1^ for 5000 cycles. The insets are charge-discharge curves of the CoMoO_4_@NiMoO_4_**·**xH_2_O//Fe_2_O_3_ ASC device collected at the first tenth and the last tenth cycles; (**e**) Charge-discharge curves of the CoMoO_4_@NiMoO_4_**·**xH_2_O//Fe_2_O_3_ ASC device collected at 3 Ag^−1^ under different bending conditions; (**f**) The Ragone plots relating power density to energy density of the supercapacitor devices and comparable previous studies.

Supercapacitors based on CoMoO_4_@NiMoO_4_·xH_2_O//Fe_2_O_3_ exhibit superb device characteristics for flexible energy storage applications. Firstly, a CV measurement is performed in a two-electrode system. Figure 6a shows the CV curves of CoMoO_4_@NiMoO_4_·xH_2_O//Fe_2_O_3_ ACS device collected at different potential voltages at a scan rate of 5 mV s^−1^. The stable potential window of the ASC can be extended to as large as 1.6 V without obvious polarization curves. Figure 6b shows the CV curves of the optimized CoMoO_4_@NiMoO_4_·xH_2_O//Fe_2_O_3_ ASC device collected at various scan rates with the potential window 0~1.6 V. All the curves show obvious pseudocapacitance features with redox peaks within 0~1.6 V, which can be attributed to the cathode and anode materials with the faradaic reactions. To further evaluate the electrochemical performance of the asymmetric cell, GCD tests of the solid-state asymmetric supercapacitor at various current densities are performed. As shown in Fig. 6c, all the charge-discharge curves show nearly symmetric behavior, confirming the excellent capacitive behavior of the device over the entire voltage range. The total specific capacitance (*C*_*t*_), which is calculated based on the total mass of active materials in the two electrodes, reaches 153.6F g^−1^ at the current density of 1A g^−1^ and still can retain 75F g^−1^ at a high current density of 15A g^−1^ (Fig. S14). Figure S14 also shows a little decrease of the specific capacitance under large current density, implying a high rate performance. The cycling life tests over 5000 cycles for CoMoO_4_@NiMoO_4_·xH_2_O//Fe_2_O_3_ were carried out at 3A g^−1^. As depicted in Fig. 6d, the CoMoO_4_@NiMoO_4_·xH_2_O//Fe_2_O_3_ ASC device exhibits a long-term electrochemical stability, and the capacitance retention after 5000 cycles is 84%. The charge-discharge curve keeps quite symmetric after 5000 cycles, indicating that there are no significant structural changes of the CoMoO_4_@NiMoO_4_·xH_2_O//Fe_2_O_3_ ASC device during the charge-discharge processes (Fig. 6d). In order to further confirm the reliability of the cycle life of the device, the cycle number of the positive electrode CoMoO_4_@NiMoO_4_·xH_2_O and negative electrode Fe_2_O_3_ also have been added (as shown in Fig. S15) to 5000 cycles at a current density of 3A g^−1^. The results indicate that the positive electrode and negative electrode have better cycle performance. The flexibility of the CoMoO_4_@NiMoO_4_·xH_2_O//Fe_2_O_3_ ACS device was performed under bending for 0°, 90° and 180° with the electrochemical test at the current density of 3A g^−1^. The compared GCD curves are indicated in Fig. 6e. The GCD profiles have almost no obvious changes, confirming that the CoMoO_4_@NiMoO_4_·xH_2_O//Fe_2_O_3_ ACS device has a remarkable mechanical flexibility. The excellent mechanical robustness and intimate interfacial contact for the multiple components demonstrate their promising utility as a flexible energy storage device.

To further demonstrate the energy and power performance of the flexible solid-state supercapacitor, Ragone plot was described based on the charge-discharge data. As shown in Fig. 6f, the energy density and power density of CoMoO_4_@NiMoO_4_·xH_2_O//Fe_2_O_3_ were calculated according to the Equations (7) and (8) the maximum specific energy as high as 41.8 Wh kg^−1^ is obtained at a current density of 1A g^−1^ with power density of 900 W kg^−1^ under the operating voltage of 1.6 V. The flexible ASC device possesses a maximum power density of 12000 W kg^−1^ at the current density of 15A g^−1^ with specific energy of 26.7 Wh kg^−1^. With an operating potential of 1.6 V, we achieve a much higher specific energy of for our asymmetric supercapacitors compared with the previous reported work^54^,^55^,^56^,^57^,^58^,^59^.

## Conclusions

In summary, we have designed and synthesized the flexible CoMoO_4_@NiMoO_4_·xH_2_O core-shell heterostructure cathode and Fe_2_O_3_ nanorods anode directly on carbon fabric. This 3D networked CoMoO_4_@NiMoO_4_·xH_2_O core-shell heterostructure facilitates fast ion diffusion and electron transfer at the electrode/electrolyte interface. The CoMoO_4_@NiMoO_4_·xH_2_O core-shell heterostructure allow the synergistic contribution of both materials leading to a better electrochemical performance. As a positive material, it exhibits excellent supercapacitor performance with a high capacitance, desirable rate performance and excellent cycling stability. Furthermore, as a negative material, Fe_2_O_3_ NWs show high specific capacitance and wide potential window compared with carbon materials. Flexible solid-state CoMoO_4_@NiMoO_4_·xH_2_O//Fe_2_O_3_ asymmetric supercapacitor is assembled by using CoMoO_4_@NiMoO_4_·xH_2_O as positive and Fe_2_O_3_ as negative electrodes, respectively. The flexible solid-state asymmetric supercapacitor with a maximum voltage of 1.6 V shows high specific energy, high power density and excellent cycling stability. Such a flexible solid-state asymmetric supercapacitor with superior performance is expected to be a promising candidate for application in energy storage devices.

### Experimental details

#### Synthesis of the CoMoO_4_ nanowire (NW) arrays on carbon fabric (CF)

Prior to the synthesis, commercial CF pieces (1 cm × 1 cm × 0.1 cm in size) were firstly ultrasonic-treated in acetone, ethanol mixture and ultrapure water, respectively. Then they were dipped in 6 M nitric acid solution and rinsed successively by ultrapure water, followed by drying in an oven at 60 °C for 5 h.

For the preparation of the CoMoO_4_ nanowires, 1.46 g of Co(NO_3_)_2_·6H_2_O and 1.21 g of Na_2_MoO_4_·7H_2_O were dissolved in 50 ml of ultrapure water under constant magnetic stirring to form a uniform light purple solution. The washed carbon fabric substrates and the light purple solution were together transferred into a 100 ml Teflon-lined stainless steel autoclave and reacted at 180 °C for 12 h. When the autoclave was cooled down to room temperature naturally, the resulting products were collected and rinsed with ultrapure water for several times. Then the products were dried in an oven at 60 °C for 12 h. Finally, to obtain CoMoO_4_ NWs, the dried samples were further annealed at 300 °C for 1 h in air.

#### Preparation of the NiMoO_4_·xH_2_O nanosheets (NSs) on carbon fabric

In a typical procedure, 0.25 g Ni(CH_3_COO)_2_·4H_2_O, 0.2 g ammonium molybdate tetrahydrate, and 0.24 g CO(NH_2_)_2_ were dissolved in 40 ml of ultrapure water and stirred constantly for 0.5 h. The solution and the cleaned CF were transferred into a 100 ml Teflon-lined stainless steel autoclave which was heated to 160 °C for 10 h. After the autoclave was cooled down to ambient, the samples were washed with ultrapure water and dried at 60 °C for 12 h. Finally, the samples were annealed at 400 °C in air for 3 h to obtain NiMoO_4_·xH_2_O NSs deposited directly on CF.

#### Preparation of the CoMoO_4_@NiMoO_4_·xH_2_O heterostructures

The as obtained CoMoO_4_ NWs on CF were immersed into the precursor solution of NiMoO_4_. Then they were together transferred to a 100 ml Teflon-lined stainless steel autoclave. The autoclave was sealed and maintained at 160 °C for 10 h and then cooled down to ambient. The as prepared CoMoO_4_@NiMoO_4_·xH_2_O core-shell heterostructures were rinsed and dried at 60 °C for 12 h. Finally, the samples were annealed at 400 °C in air for 3 h to obtain CoMoO_4_@NiMoO_4_ deposited on CF.

#### Preparation of the Fe_2_O_3_ nanorods (NRs) on carbon fabric

The Fe_2_O_3_ NRs were prepared as follows: 1.08 g FeCl_3_·6H_2_O and 0.56 g Na_2_SO_4_ were dissolved in 80 ml of ultrapure water and constantly stirred for 0.5 h. The mixed solution and the cleaned CF were transferred together into a 100 ml Teflon-lined stainless steel autoclave which was heated to 120 °C for 8 h. After the autoclave was cooled to ambient naturally, the samples washed with distilled water and dried at 60 °C for 12 h. Finally, the samples were annealed at 400 °C in air for 3 h to obtain Fe_2_O_3_ NRs. Na_2_SO_4_ was used as the structure-directing agent to facilitate the uniform growth of 1D structures^60^.

#### Materials Characterizations

The microstructure and morphology were characterized by Scanning electron microscopy (SEM, Hitachi S-4800, at an acceleration voltage of 20 kV) and Transmission electron microscopy (TEM, JEOL JEM-2010). The phase structures of the as-prepared products were characterized by X-ray diffraction (XRD, Rigaku D/max-rB, Cu Kα radiation, λ = 0.1542 nm, 40 kV, 100 mA). Brunauer-Emmett-Teller (BET) analysis was carried out to evaluate the surface area and pore size distribution of the as prepared products. Surface Area Analyzer (NOVA2000E) was used to measure N_2_-sorption isotherm.

#### Electrochemical measurements

The electrochemical measurements were firstly conducted in a three-electrode form. CoMoO_4_@NiMoO_4_·xH_2_O/CF electrodes were used as the working electrode. A platinum foil (1 cm × 4 cm) acted as the counter electrode and a saturated calomel electrode (SCE) acted as the reference electrode. 2.0 M KOH aqueous solution served as the electrolyte. The electrochemical measurements were carried out on a CHI 660 C electrochemistry workstation (Shanghai, China). Cyclic voltammetry (CV) tests were conducted in a potential range of −0.2~0.6 V (versus SCE) at different sweep rates of 5~100 mV s^−1^. The constant current charge/discharge tests were carried out at various current densities within a potential range of 0~0.5 V (versus SCE), and the cycling behavior was characterized up to 3000 cycles (at a current density of 3A g^−1^) and 10000 cycles (at a current density of 5A g^−1^), respectively. Electrochemical impedance spectroscopy (EIS) was performed to determine the capacitive performance at open circuit voltage with a frequency range of 0.01~10^5^ Hz. The CV curves and charge-discharge curves of Fe_2_O_3_ NRs were also tested. These electrochemical measurements were performed in a three-electrode system.

#### Assembly of the solid-state asymmetric supercapacitor (ASC)

Solid-state ASC was fabricated using CoMoO_4_@NiMoO_4_·xH_2_O electrode as the positive electrode and Fe_2_O_3_ NRs electrode as the negative electrode. The CoMoO_4_@NiMoO_4_·xH_2_O electrode was resized to 1.0 cm × 1.0 cm in size with an average mass loading of 1.8 mg cm^−2^. The Fe_2_O_3_ NRs electrode was resized the same size with the mass loading of 2.3 mg cm^−2^. Then, the polyvinyl alcohol (PVA)/KOH gel electrolyte was prepared by mixing as-prepared 6 g PVA with 5.6 g KOH in 50 ml of deionized water and heated at 80 °C under stirring for 3 h until it became homogeneously clear. The electrodes and the separator were soaked in the gel for 5 min, then taken out from the gel, and assembled together. The device was placed in the air for 24 h and became solid. Afterward, the ASC device was assembled by sandwiching PVA/KOH gel electrolyte film between the Fe_2_O_3_/CF and CoMoO_4_@NiMoO_4_·xH_2_O/CF electrodes under mechanical stress. The specific capacitance, energy density, and power density of the ASC were all calculated based on the total mass of both negative and positive electrodes excluding the weights of current collectors. The thickness of the as-prepared solid-state ASC was measured to be about 1.15~1.34 mm. All electrochemical tests of the ASC device were performed in a two electrode configuration at ambient temperature.

The following equations were used to calculate the specific capacitance *C*_*s*_ (F g^−1^), specific energy *E* (Wh kg^−1^) and power density *P* (W kg^−1^):


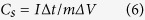



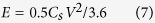



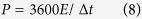


where *I* (A) represents the discharge current, Δ*t*(s) is the discharge time, *ΔV* (V) is the potential drop during discharge process, *m* (g) is the mass of the active materials, *S* is the enclosed area of the discharge curve and coordinate axis, and *U* (V) is the potential window.

## Additional Information

**How to cite this article**: Wang, J. *et al*. Assembly of flexible CoMoO_4_@NiMoO_4_·xH_2_O and Fe_2_O_3_ electrodes for solid-state asymmetric supercapacitors. *Sci. Rep.* **7**, 41088; doi: 10.1038/srep41088 (2017).

**Publisher's note:** Springer Nature remains neutral with regard to jurisdictional claims in published maps and institutional affiliations.

## Supplementary Material

Supporting Information
