# Supplementary material for: Assembly of flexible CoMoO4@NiMoO4·xH2O and Fe2O3 electrodes for solid-state asymmetric supercapacitors
Source: Sci Rep. 2017 Jan 20;7:41088. doi: 10.1038/srep41088 (PMC5247727; doi:10.1038/srep41088)
Supplement: Supporting Information [file srep41088-s1.pdf]

Supporting information for

Assembly of flexible $CoMoO_4$@$NiMoO_4 \cdot xH_2O$ and Fe_2O_3 electrodes for solid-state asymmetric supercapacitors

Jing Wang‡ [a], Leipeng Zhang‡ [b], Xusong Liu [a], Xiang Zhang [b], Yanlong Tian [b], Xiaoxu Liu [a,c], Jiupeng Zhao [a,], Yao Li[b,]**

[a] *MIIT Key Laboratory of Critical Materials Technology for New Energy Conversion and Storage, School of Chemistry and Chemical Engineering, Harbin Institute of Technology, Harbin 150001, PR China*

[b] *Center for Composite Materials Harbin Institute of Technology, Harbin 150001, PR China*

[c] *Heilongjiang University of Science and Technology, Harbin 150022, PR China*

‡ *These two authors contributed equally to this work.*

[*] To whom correspondence should be addressed：*E-mail: jpzhao@hit.edu.cn yaoli@hit.edu.cn*

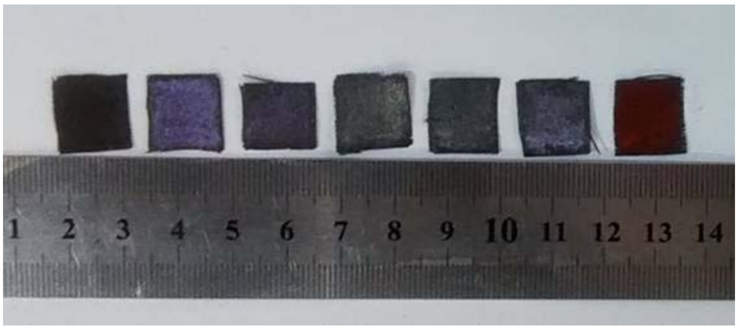

Fig. S1 The optical images of the as prepared electrodes. From the left to right in turn are carbon fabric, $CoMoO_4$ precursor on CF, $CoMoO_4$ on CF, $NiMoO_4 \cdot xH_2O$ precursor on CF, $NiMoO_4 \cdot xH_2O$ on CF, $CoMoO_4$@$NiMoO_4 \cdot xH_2O$ core-shell heterostructures and Fe_2O_3 on CF, respectively.

.

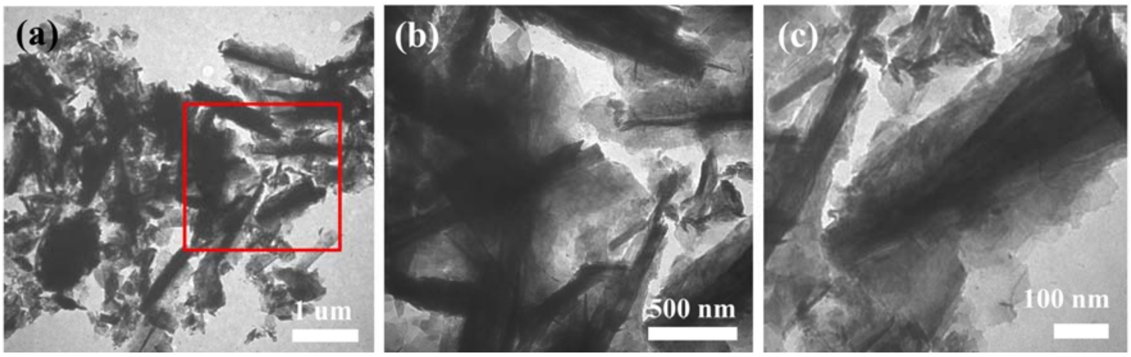

Fig.S2 TEM images of the $CoMoO_4$@$NiMoO_4 \cdot xH_2O$ at different magnifications. The labeled in the red zone is selected for different magnifications.

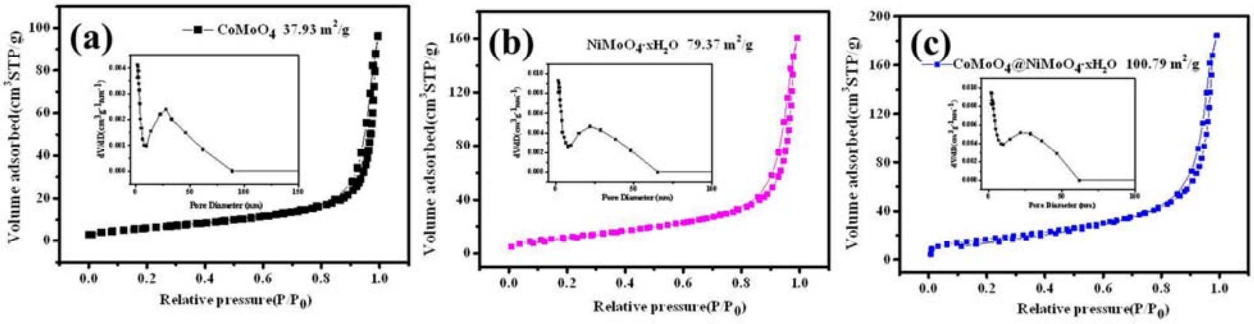

Fig.S3 N_2 adsorption and desorption isotherms of (a) $CoMoO_4$ NWs; (b) $NiMoO_4 \cdot xH_2O$ NSs; (c) $CoMoO_4$@$NiMoO_4 \cdot xH_2O$.

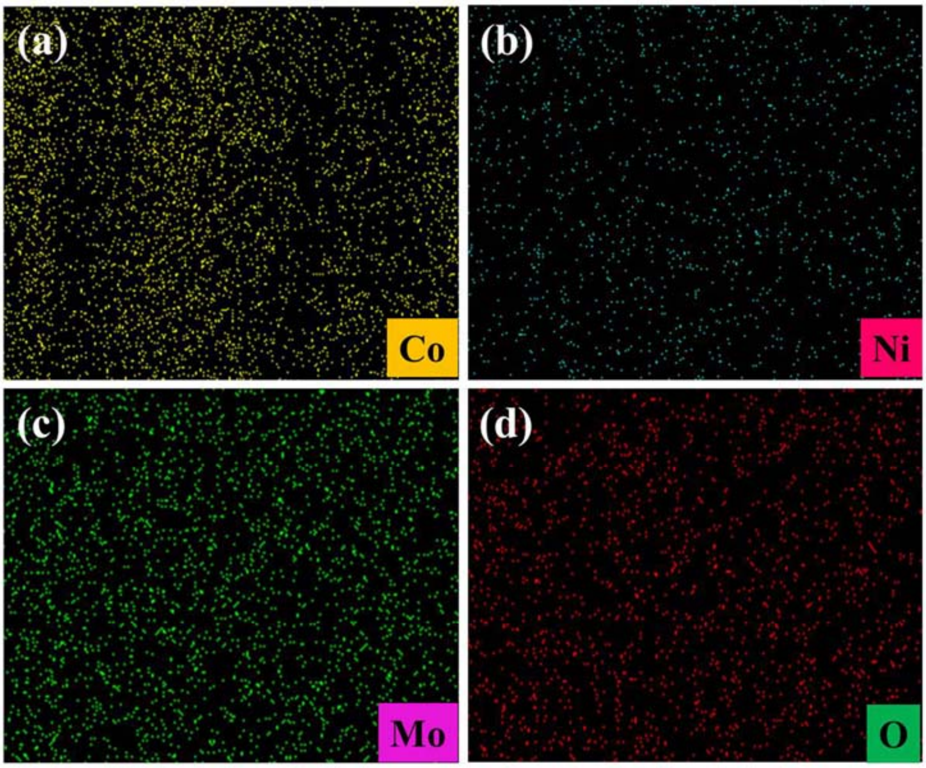

Fig. S4(a-d) SEM mapping images of Co, Ni, Mo and O elements, respectively.

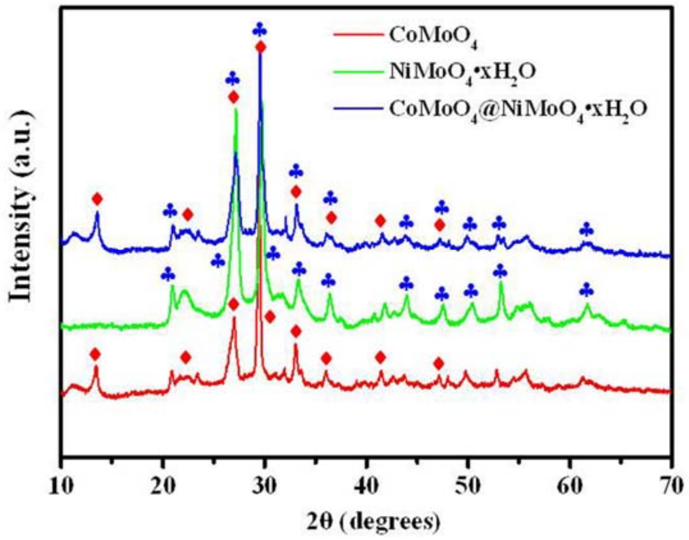

Fig.S5 XRD patterns of $CoMoO_4$ NWs, $NiMoO_4{\cdot}xH_2O$ NSs, and $CoMoO_4@NiMoO_4{\cdot}xH_2O$ core-shell heterostructures.

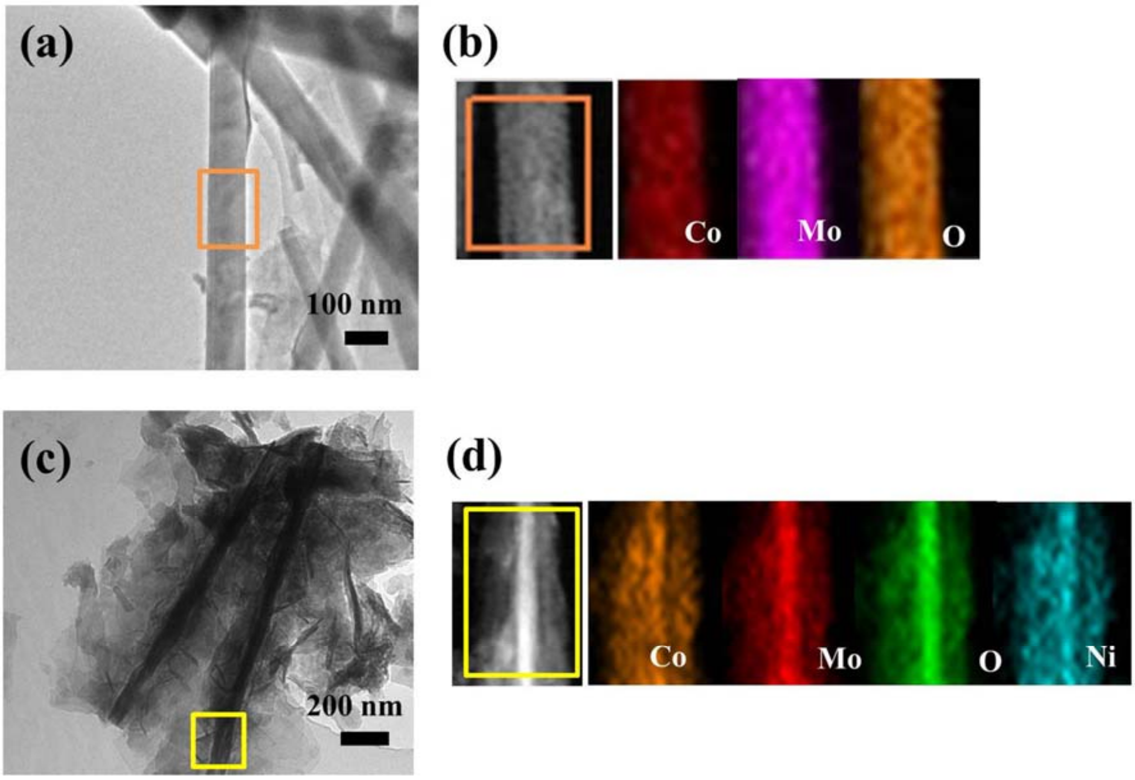

Fig. S6 (a) Typical TEM image of the $CoMoO_4$ NWs. The labeled in the orange zone is selected for mapping images of (b) Co element, Mo element and O element. (c) Typical TEM image of the $CoMoO_4$@$NiMoO_4 \cdot xH_2O$ core-shell heterostructures. The labeled in the yellow zone is selected for mapping images of (d) Co element, Mo element, O element and Ni element.

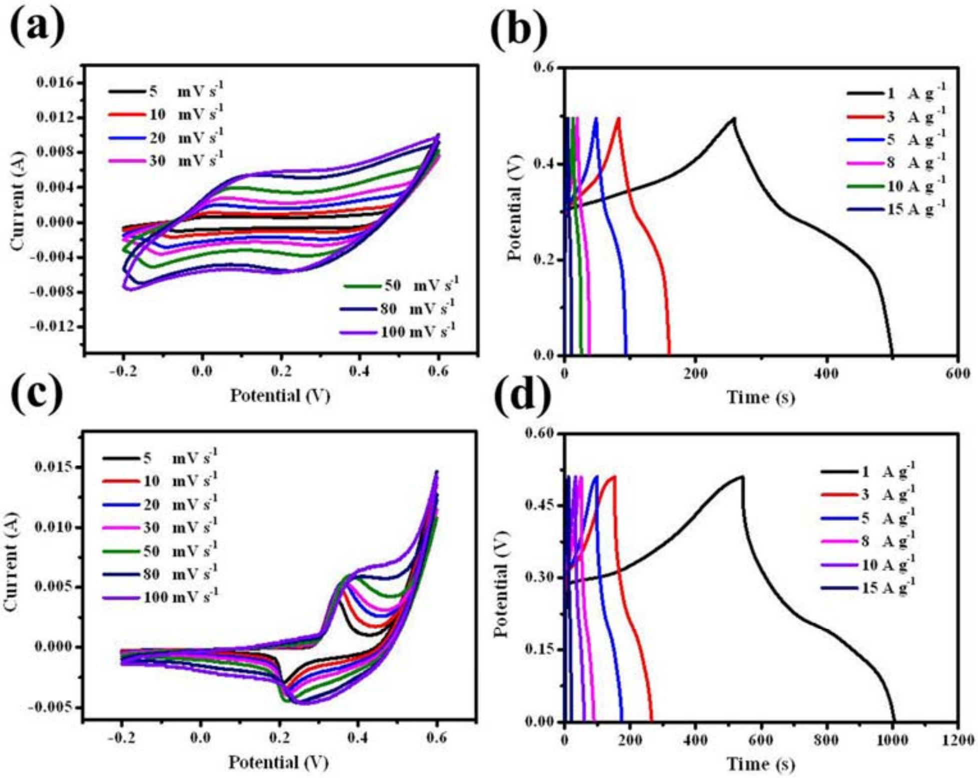

Fig. S7 (a, c) Cyclic voltammograms of $CoMoO_4$ NWs and $NiMoO_4{\cdot}xH_2O$ NSs electrodes obtained at different scan rates, respectively; (b, d) Charge/discharge curves of $CoMoO_4$ NWs and $NiMoO_4{\cdot}xH_2O$ NSs electrodes at different current densities, respectively.

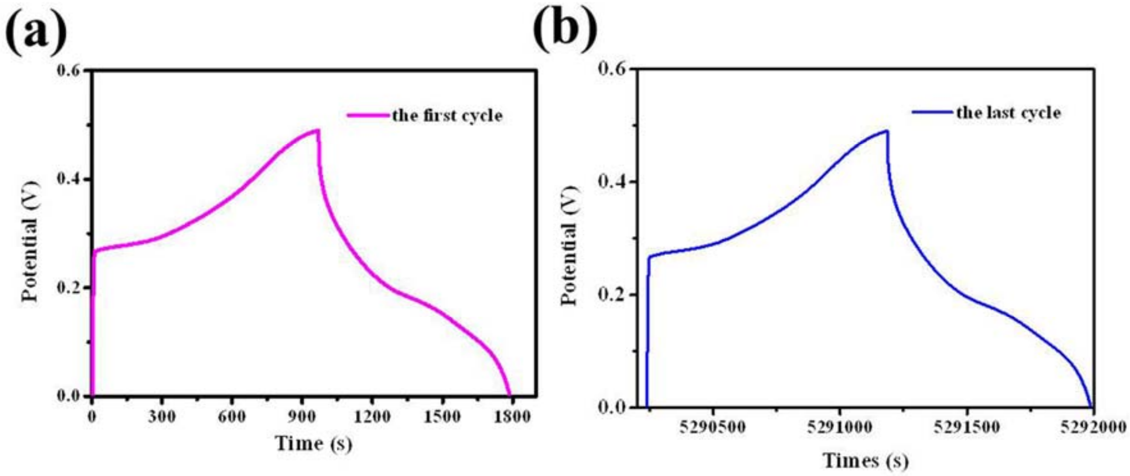

Fig. S8 (a), (b) Charge/discharge curves of the $CoMoO_4$@$NiMoO_4{\cdot}xH_2O$ electrode at the first cycle and the last cycle at the current density of 1 A g^{-1} for 3000 cycles, respectively.

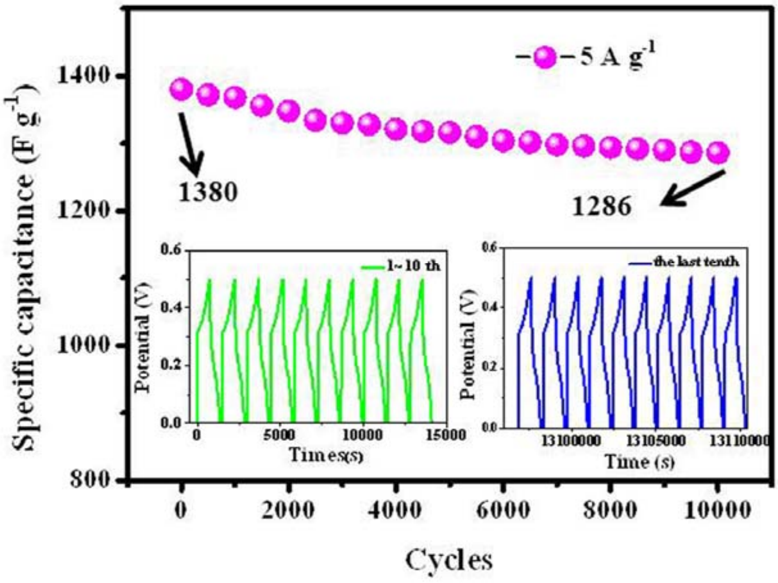

Fig. S9 Cycle performance of the $CoMoO_4@NiMoO_4{\cdot}xH_2O$ electrode at the current density of 5 A g^{-1} for 10000 cycles. The insets are the charge-discharge curves for the first tenth and the last tenth cycles, respectively.

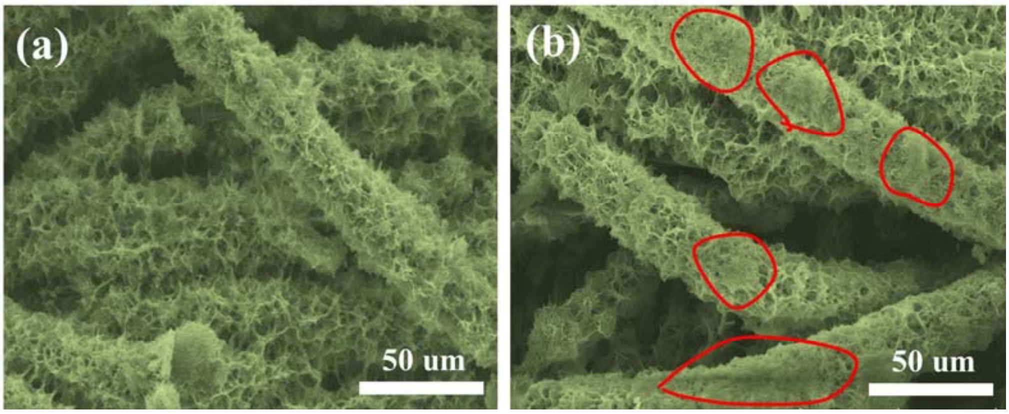

Fig. S10 SEM image of the $CoMoO_4$@$NiMoO_4{\cdot}xH_2O$ electrode before and after 10000 cycles at a current density of 5 A g^{-1}.

Table S1. Comparison of specific capacitances and cycling performance of the reported $CoMoO_4$ or $CoMoO_4$ oxides based electrodes and the present work.

Electrode materials	Current density (A g^{-1})	Capacitance (F g^{-1})	Ref
$CoMoO_4$-$NiMoO_4$ nanotubes	1	751	[1]
$CoMoO_4$-$NiMoO_4$•xH_2O bundles	1	1039	[2]
$MnMoO_4$/$CoMoO_4$ heterostructured nanowires	3	134.7	[3]
$CoMoO_4$/graphene composites	1	394.5	[4]
$NiMoO_4$@$CoMoO_4$ hierarchical nanospheres	6	812	[5]
Polyaniline-wrapped 1D $CoMoO_4$•$0.75H_2O$ nanorods	1	380	[6]
Hierarchical 3-dimensional $CoMoO_4$ nanoflakes	1	352	[7]
$NiMoO_4$ Nanorods	1<br>10	974.4<br>821.4	[8]
Hierarchical nanosheet-based $NiMoO_4$ nanotubes	1	864	[9]
graphene decorated with 1D $NiMoO_4$·nH_2O nanorods	5	367	[10]
$NiMoO_4$·H_2O nanoclusters	1	680	[11]
$NiMoO_4$ · xH_2O nanorods	1	1131	[12]
$CoMoO_4$@$NiMoO_4$•xH_2O core-shell heterostructure	10<br>5<br>3<br>1	1050<br>1380<br>1470<br>1582	This work

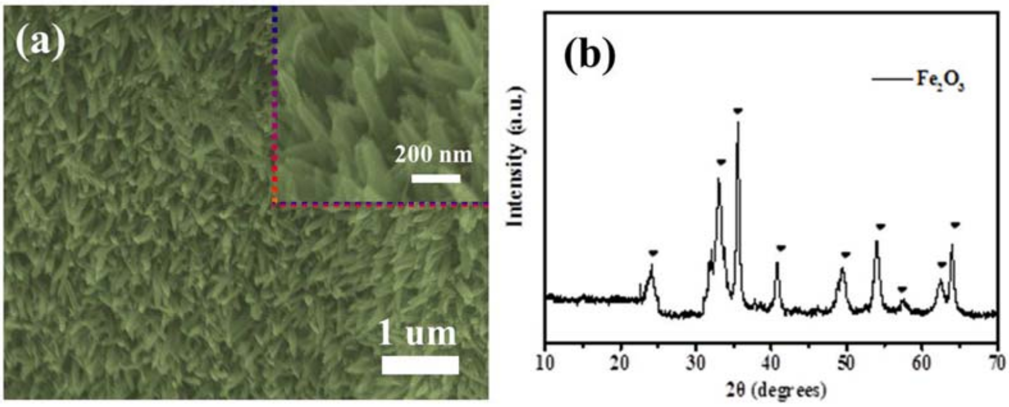

Fig. S11 (a) SEM image of the as prepared Fe_2O_3NRs; (b) XRD pattern of the Fe_2O_3 NRs.

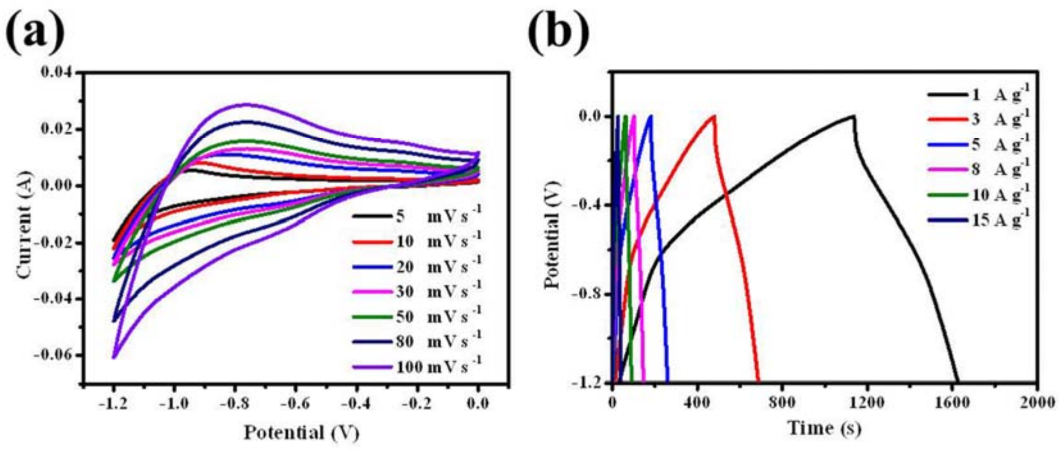

Fig. S12 Cyclic voltammograms of the Fe_2O_3 NRs; (b) Charge/discharge curves of the Fe_2O_3 NRs electrode at different current densities.

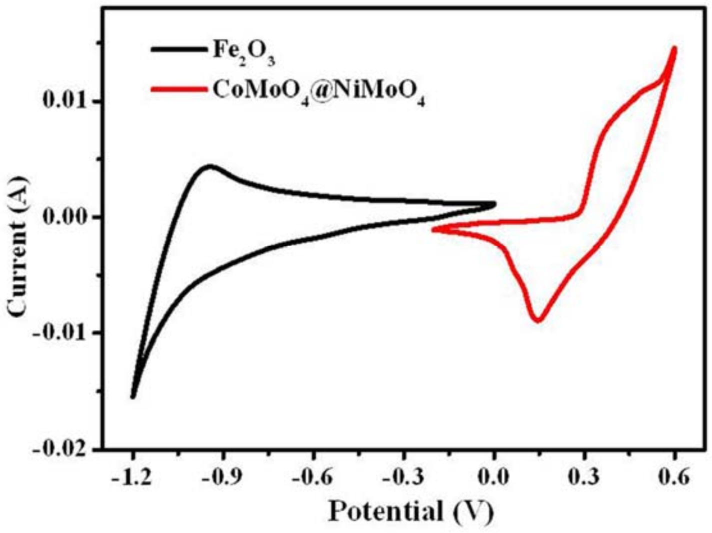

Fig. S13 CV curves of the $CoMoO_4@NiMoO_4 \cdot xH_2O$ and Fe_2O_3 electrodes performed in a three-electrode cell in 2M KOH electrolyte at a scan rate of 5 mV s^{-1}.

The mass of positive and negative is different. For the supercapacitor, the charge balance follows the relationship $q^+ = q^-$, where q^+ and q^- represent the charge stored in positive electrode and negative electrode, respectively. The q of each electrode depends on the specific capacitance (Cs), the potential range of the charge/discharge tests(ΔV), and the mass of the electrode materials according to the following equation:

$$q = Cs \times \Delta V \times m \qquad (1)$$

when $q^+=q^-$, the masses of the positive electrode (m^+) and negative electrode (m^-) will follow the equation:

$$\frac{m^+}{m^-} = \frac{C^- \times \Delta V^-}{C^+ \times \Delta V^+} \qquad (2)$$

The specific capacitances of the Fe_2O_3 and $CoMoO_4$@$NiMoO_4 \cdot xH_2O$ are 516.7 F g^{-1} and 1582 F g^{-1} at the same current density of 1 A g^{-1}, respectively. The potential range of the charge/discharge tests for Fe_2O_3 and $CoMoO_4$@$NiMoO_4 \cdot xH_2O$ electrodes are 1.2 V and 0.5 V, respectively. On the basis of the specific capacitance values and the potential windows for the Fe_2O_3 and $CoMoO_4$@$NiMoO_4 \cdot xH_2O$ electrodes, the optimal mass ratio should be $m^+/m^- = 1/1.3$ according to the equation (2) for assembled the asymmetric supercapacitor device. Herein, the masses of the as-prepared Fe_2O_3 and $CoMoO_4$@$NiMoO_4 \cdot xH_2O$ electrodes are 2.3 mg cm^{-2} and 1.8 mg cm^{-2}., respectively. The optimal mass ratio is close to 1.3.

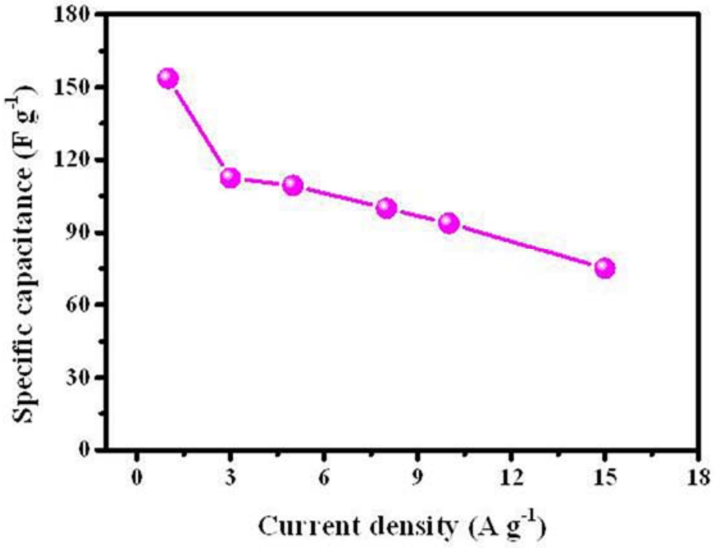

Fig. S14 Plot of the current density against the specific capacitance of the $CoMoO_4$@$NiMoO_4 \cdot xH_2O$//Fe_2O_3 ACS device.

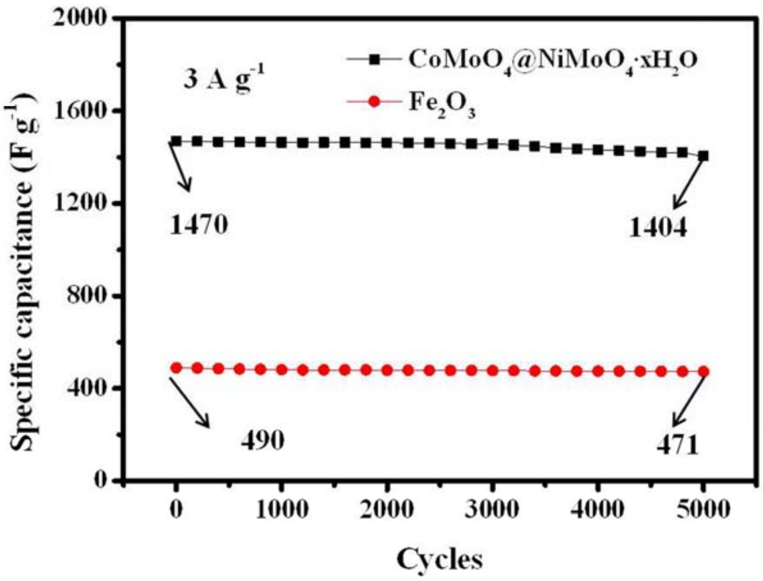

Fig. S15 Cycle performance of positive electrode ($CoMoO_4$@$NiMoO_4 \cdot xH_2O$) and the cyclability of negative electrode (Fe_2O_3) at the current density of 5 A g^{-1} for 5000 cycles.

References

[1] Yang, Q., Lin, S. Y. Rationally designed nanosheet-based $CoMoO_4$-$NiMoO_4$ nanotubes for high-performance electrochemical electrodes. *RSC Advances*, **6**, 10520-10526 (2016).

[2] Liu, M. C. et al. Design and synthesis of $CoMoO_4$-$NiMoO_4$• x H_2O bundles with improved electrochemical properties for supercapacitors. *J. Mater. Chem. A* **1**, 1380-1387 (2013).

[3] Mai, L. Q. et al. Hierarchical $MnMoO_4$/$CoMoO_4$ heterostructured nanowires with enhanced supercapacitor performance, *Nat. Commun.* **2**, 381(2011).

[4] Xia, X. F., Lei, W., Hao, Q. L., Wang, W. J., Wang, X. One-step synthesis of $CoMoO_4$/graphene composites with enhanced electrochemical properties for supercapacitors. *Electrochimica Acta* **99,** 253-261(2013).

[5] Zhang, Z. et al. Facile hydrothermal synthesis of $NiMoO_4$@$CoMoO_4$ hierarchical nanospheres for supercapacitor applications. *Phys.Chem.Chem.Phys.* **17**, 20795-20804 (2015).

[6] Mandal,M., Ghosh, D., Giri, S., Shakirb, I., Das, C. K. Polyaniline-wrapped 1D $CoMoO_4$· 0.75 H_2O nanorods as electrode materials for supercapacitor energy storage applications. *RSC Adv.***4** 30832-30839 (2014).

[7] M, Li., Xu,S., Cherry, C. Hierarchical 3-dimensional $CoMoO_4$ nanoflakes on macroporous electrically conductive network with superior electrochemical performance, *Journal of Materials Chemistry A* **3**, 13776-13785(2015).

[8] Cai, D. et al. Comparison of the electrochemical performance of $NiMoO_4$ nanorods and hierarchical nanospheres for supercapacitor applications. *ACS applied materials & interfaces*, **5**, 12905-12910(2013).

[9] Yin, Z. Hierarchical nanosheet-based $NiMoO_4$ nanotubes: synthesis and high supercapacitor performance. *Journal of Materials Chemistry A*, 3,739-745 (2015).

[10] Ghosh, D., Giri, S., Das, C. K. Synthesis, characterization and electrochemical performance of graphene decorated with 1D $NiMoO_4 \cdot nH_2O$ nanorods. *Nanoscale,* **5**, 10428-10437(2013).

[11] Wan, H. et al. Rapid microwave-assisted synthesis NiMoO 4· H 2 O nanoclusters for supercapacitors[J]. Materials Letters, 2013, 108: 164-167.

[12] Liu, P. et al. Facile synthesis and characterization of high-performance $NiMoO_4 \cdot xH_2O$ nanorods electrode material for supercapacitors. *Ionics*, **21,** 2797-2804(2015).
